# Alcohol-induced psychosis and delirium tremens: a comparison with alcohol dependence on demographic characteristics, mortality, and morbidity

**DOI:** 10.1186/s12888-025-06753-z

**Published:** 2025-03-25

**Authors:** Jørgen G. Bramness, Eline Borger Rognli, Anne Høye, Ina H. Heiberg

**Affiliations:** 1 Institute of Clinical Medicine, UiT The Arctic University of Norway, Tromsø, Norway; 2 Section for Clinical Addiction Research, Oslo University Hospital, Oslo, Norway; 3 Norwegian National Advisory Unit on Concurrent Substance Abuse and Mental Health Disorders, Innlandet Hospital Trust, Brumunddal, Norway; 4 Department of Drugs and Tobacco, Norwegian Institute of Public Health, Oslo, Norway; 5 Mental Health and Substance Abuse Clinic, University Hospital North Norway, Tromsø, Norway; 6 Center for Clinical Documentation and Evaluation, Tromsø, Norway

**Keywords:** Alcohol use disorder, Alcohol dependence, Alcohol induced psychosis, Delirium tremens, Morbidity, Mortality

## Abstract

**Objectives:**

The study aim was to compare patients with alcohol-induced psychosis (AIP) and delirium tremens (DT) with patients with alcohol dependency (AD) only. Using data from Norwegian Patient Registry (NPR) we investigated demographic characteristics, mortality, and physical and mental health comorbidities, among individuals with AIP or DT compared with AD patients.

**Methods:**

Data from NPR was used to create a cohort of patients aged 20–79 diagnosed with either AIP, DT or AD, from 2009 to 2015. If patients received more than one of these diagnoses, AIP and DT were prioritized. For patients with both AIP and DT, the earliest diagnosis took priority, except when the diagnoses were assigned simultaneously, when DT was prioritized. Data on comorbidities were taken from NPR, while cause of death was obtained from the Norwegian Cause of Death Registry. Estimates were compared using chi-square test and the Kruskal-Wallis test with Bonferroni adjustments for multiple testing. Mortality was analysed using Cox regression models and by calculating standardized mortality ratios, adjusting for age and gender.

**Results:**

The cohort included 33 107 patients diagnosed with AD, 1 784 with DT, and 700 with AIP. AIP patients were the youngest. DT patients displayed significantly higher mortality rates, with an annual rate of 8.0%, and generally increased comorbidity rates. AIP patients showed significantly higher rates of schizophrenia spectrum disorders compared to both AD and DT patients, highlighting a potential link between AIP and psychotic disorders.

**Conclusion:**

This study reveals that patients with DT experience higher morbidity and mortality rates compared to those with AIP and AD. AIP patients did not show increased all-cause or cause-specific mortality compared to AD patients across a variety of causes. Notably, AIP seemed to be more closely linked to comorbid schizophrenia spectrum disorders than AD and DT patients. The findings underscore the complexities of AIP in relation to schizophrenia and highlight significant differences in health outcomes among the three patient groups.

## Introduction

Psychotic disorders may be related to heavy alcohol use and especially alcohol dependency (AD) (ICD-10 diagnosis F10.2). The psychotic phenomena that more or less directly follow extensive alcohol use or AD vary in type and possibly in origin. Korsakoff’s psychosis (F10.6) is probably caused by lack of thiamine (vitamin B1) [1]. Additionally, there are conditions such as delirium tremens (DT) (ICD-10 code F10.4) and alcohol-induced psychosis (AIP) (ICD-10 code F10.5).

AIP is a psychotic condition triggered by alcohol use, and it is less commonly associated with withdrawal. AIP is characterized by psychotic symptoms such as auditory hallucinations, and persecutory delusions, and a near to normal level of consciousness [2]. Some authors suggest that differentiating AIP from DT can be challenging; however, the clear sensorium in AIP is emphasized as an important distinguishing factor [3]. Alcoholic hallucinosis (F10.52) is a subcategory of AIP [4]. A recent review [4] reveals great variation in the prevalence of alcoholic hallucinosis in AD patients, ranging from approximately 0.5% [5, 6], to 4% [7], and up to around 10% [8, 9]. In an observational study from the early 2000 it was found that even if the share of alcohol use disorder (AUD) patients entering hospitals with AIP is high, the trend was decreasing [4]. This downward trend is also seen in a Scandinavian study from 2000 to 2016 [10].

DT can occur after reducing or stopping high and prolonged alcohol use. It is characterized by sweating, hypertension, tachycardia, tremor, insomnia, and anxiety [11, 12], visual hallucinations, impaired consciousness and generalized seizures [11, 13]. Untreated, the acute DT mortality is up to 35% [14], while correct treatment can reduce mortality to close to zero [15]. DT has an estimated prevalence range in people with AUD from 5 to 12% [16–19]. This is confirmed by a recent study from our group [20].

Only a few studies have examined the relationship between AIP and DT. Some view them as different manifestations of alcohol withdrawal [21], while others view them as qualitatively different entities [2]. Quite a few studies do not properly distinguish between the two disorders in their investigation of the phenomena [2]. Aside from the important features of impaired vs. clear sensorium and visual vs. auditory hallucinations, there are quite a few similarities between the two [3]. An investigation of 25 individuals with AIP and 14 participants with DT found considerable medical comorbidity for the group, and during an eight-year follow-up, 37% had died [7]. In spite of this high mortality, and although both DT and AIP have been described as risk factors for mortality in people with AUD [22], to our knowledge, no studies have compared mortality in DT versus AIP. Also, we have found no studies comparing the co-morbidities related to the two diagnoses.

A feature that has been extensively discussed is the relationship between AIP and schizophrenia, with views differing from AIP being a schizophrenia spectrum disorder, largely unrelated to the use of alcohol, to it being a condition distinct from schizophrenia, despite symptom similarities [2, 3, 23, 24]. Our recent study indicates that one in ten AIP patients over a six-year period are re-diagnosed with schizophrenia spectrum disorder [25]. This suggests that even if AIP and schizophrenia are separate diagnoses and possibly separate phenomena, they are related.

The present study was not preregistered and is exploratory and descriptive it its nature. We utilized data from Norwegian national patient registries to compare demographic characteristics, physical and mental health comorbidities, and mortality rates among individuals with AIP to patients with AD only. For comparison we also compared DT patients to patients with AD only.

## Methods

### Setting and participants

The Norwegian Patient Registry (NPR) is a comprehensive national database that includes all instances of publicly funded specialized inpatient or outpatient treatment throughout Norway. In the country, most specialized healthcare services receive public funding. The study cohort consists of all individuals aged 20 to 79 who were recorded in the NPR at least once between January 1st, 2009, and December 31st, 2015, with either a primary or secondary diagnosis of AD, DT, or AIP. If a patient had multiple diagnoses, priority was given to DT and AIP over AD; among those diagnosed with both DT and AIP, the condition that was diagnosed first received precedence, unless both were diagnosed simultaneously, in which case DT was prioritized. Diagnoses in the NPR are typically recorded based on clinical documentation provided by attending physicians.

Patients were tracked prospectively from the date of their first diagnosis of either DT, AIP, or AD (the index episode), until either December 31st, 2015, or the date of censoring (defined as death, emigration from Norway, or December 31st of the year they turned 79, whichever came first). Those patients who were already hospitalized on January 1st, 2009, were monitored starting from that date (*n* = 426). Additional data regarding the patients’ registered binary gender and age at the time of the index episode were sourced from the National Population Register. The unique 11-digit national personal identifier, noted during each treatment episode, facilitated the tracking of individuals across the NPR and enabled the linkage of patient data to the Norwegian Cause of Death Registry (NCDR).

### Measures

#### Mortality

Information regarding emigration or death of cohort participants during the observation period was sourced from Statistics Norway, while the causes of death were retrieved from NCDR. All-cause mortality was examined through three metrics: (i) the percentage of patients who passed away within the first month following the index episode; (ii) the percentage who died over the entire observation period; and (iii) the crude annual mortality rate, defined as the number of deaths divided by the total person-years under observation from the index episode until censoring or December 31st, 2015. For every reported death, we collected cause of death information from the NCDR, which encompasses 98% of all deaths in Norway [26]. Causes of death were classified based on the ICD-10 coding system. Natural causes (A00–R99) were categorized into cardiovascular disease (I00–I99), respiratory disease (J00–J99), cancer (C00–C97), and other natural causes. Unnatural causes of death (V01–Y98) were classified into poisoning (X40–X49), suicide (X60–X84 and Y87.0), and other unnatural causes, which included accidents (V01-V89).

Data reflecting the annual number of deaths, stratified by gender and five-year age groups within the general population, was obtained from the Norwegian Institute of Public Health [27]. Additionally, annual demographic data for the age groups of 20 to 79 years during the period from 2009 to 2015 was sourced from Statistics Norway.

#### Alcohol-related comorbidity

For participants in the cohort, we gathered data from NPR regarding ICD-10 diagnoses related to any of the following emergencies, diseases, or injuries commonly associated with AUD patients: acute alcohol intoxication (F10.0), alcoholic liver disease (K70), alcohol-induced chronic pancreatitis (K86.0), polyneuropathies (G60–G64), dementia (F00–F03, F05.1, G31.1), and head injuries (S00–S09) [28, 29]. The psychiatric comorbidities included in this study encompassed all schizophrenia spectrum disorders (F2), with a specific focus on schizophrenia (F20). We considered the presence of one or more of these comorbidities to be indicative of a more severe form of AD. For each of these comorbidities, we analyzed the proportion of patients diagnosed (at least once) during the two years prior to the index episode and in the first two years following the index episode (up to December 31st, 2015, or until censoring), comparing the results across the three patient groups: AD, AIP, and DT.

### Data analysis

**Fig. 1:**
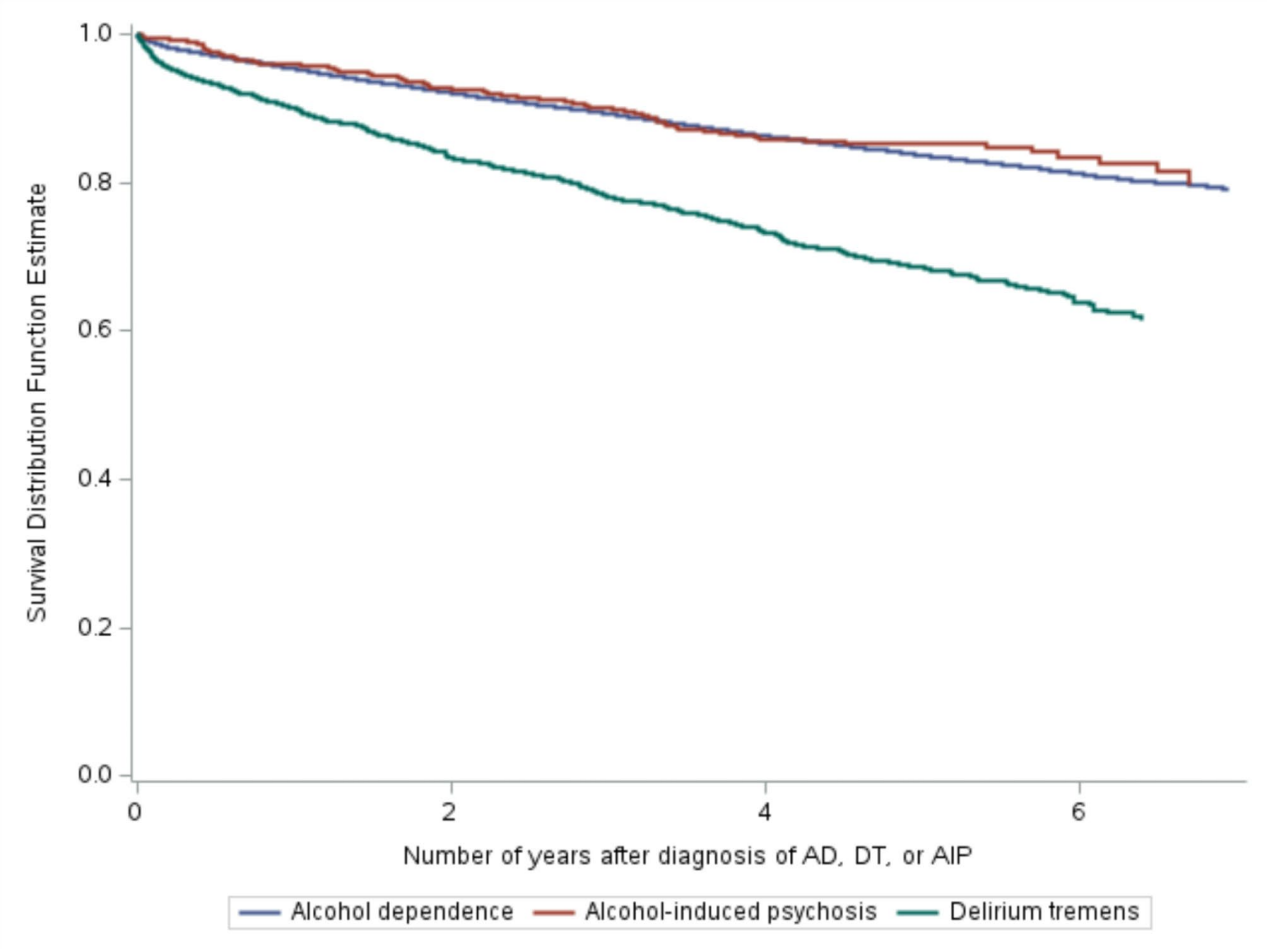
Kaplan-Meier curves displaying the survival probability for patients with alcohol dependency (AD) (blue curve), delirium tremens (DT) (green curve), or alcohol-induced psychosis (AIP) (red curve) after the index episode

We conducted a comparison of the three patient groups with respect to gender, age, crude mortality rates (Table 1), and alcohol-related comorbidity. Chi-square tests were used to assess the overall statistical significance of categorical variables, with post hoc pairwise comparisons using Fisher’s exact tests and Bonferroni adjustments to account for multiple testing. The Kruskal-Wallis test was employed to evaluate overall statistical significance for continuous variables, followed by Dunn’s test with Bonferroni adjustments [30]. A two-sided p-value of less than 0.05 was deemed statistically significant. The Kaplan-Meier estimator was utilized to derive the survival functions for the three groups (Fig. 1). Additionally, we calculated standardized mortality ratios (SMRs) with 95% confidence intervals (CIs) for both all-cause and cause-specific mortality (Table 2). The SMR was determined by dividing the observed number of deaths by the expected number of deaths in the study group, calculated by applying the age- gender-, and calendar year specific mortality rates in the general population to the matching strata. We used the approximate Poisson method to compute the standards errors and CIs, with an exponential (log-based) function approach to estimate the CIs.

**Table 1:** Demographic characteristics and measures of crude mortality by patient group

		Alcohol dependency (AD)	Delirium tremens (DT)	Alcohol-induced psychosis (AIP)	*p*-value^a^	Post hoc tests
		*N* = 33 107	*N* = 1 784	*N* = 700		
Age at inclusion	Mean (SD)	48.7 (13.9)	56.0 (11.3)	46.3 (14.3)	< 0.001	DT > AD > AIP
Male	*N* (%)	23 649 (71.4)	1 433 (80.3)	503 (71.9)	< 0.001	DT > AD/AIP
Mortality						
First month after the index episode	*N* (%)	349 (1.1)	45 (2.5)	3 (0.4)	< 0.001	DT > AD/AIP
Throughout the observation period	*N* (%)	4 465 (13.5)	433 (24.3)	81 (11.6)	< 0.001	DT > AD/AIP
Annual mortality rate	%	3.7	8.0	3.4		

^a^ Significance tested with the chi-square or Kruskal-Wallis tests

**Table 2:** Distribution of causes of death and standardized mortality ratios (SMR) for all-cause and cause-specific mortality over the study period by patient group; AD, DT, and AIP

	Alcohol dependence (AD) *N* = 33 107		Delirium tremens (DT) *N* = 1 784		Alcohol-induced psychosis (AIP) *N* = 700		
	Died *n* (%)	SMR (95% CI)	Died *n* (%)	SMR (95% CI)	Died *n* (%)	SMR (95% CI)	Post hoc test
All-cause mortality	4 465 (13.5)	6.9 (6.7–7.1)	433 (24.3)	9.7 (8.9–10.7)	81 (11.6)	7.1 (5.7–8.8)	DT > AD/AIP
Natural causes of death	3 461 (10.5)	6.1 (5.9–6.3)	345 (19.3)	8.6 (7.7–9.5)	54 (7.7)	5.4 (4.1-7.0)	DT > AD/AIP
Cardiovascular disease	765 (2.3)	5.3 (5.0-5.7)	78 (4.4)	7.4 (5.9–9.2)	9 (1.3)	3.6 (1.9-7.0)	DT > AD
Respiratory disease	436 (1.3)	9.6 (8.7–10.5)	46 (2.6)	13.2 (9.9–17.6)	9 (1.3)	11.1 (5.8–21.4)	n.s.
Cancer	780 (2.4)	3.1 (2.9–3.3)	52 (2.9)	3.0 (2.3–3.9)	9 (1.3)	2.0 (1.0-3.8)	n.s.
Other natural causes	1 480 (4.5)	13.6 (12.9–14.3)	169 (9.5)	22.5 (19.4–26.2)	27 (3.9)	14.0 (9.6–20.4)	DT > AD/AIP
Unnatural causes	897 (2.7)	15.3 (14.3–16.3)	80 (4.5)	26.6 (21.4–33.1)	22 (3.1)	19.5 (12.8–29.6)	DT > AD
Poisonings	347 (1.0)	28.5 (25.7–31.7)	30 (1.7)	59.8 (41.8–85.5)	9 (1.3)	37.1 (19.3–71.3)	DT > AD
Suicide	283 (0.9)	13.9 (12.4–15.7)	22 (1.2)	23.9 (15.7–36.2)	7 (1.0)	17.5 (8.3–36.7)	DT > AD
Other unnatural causes	267 (0.8)	10.5 (9.3–11.8)	28 (1.6)	18.3 (12.6–26.4)	6 (0.9)	12.6 (5.7–28.1)	DT > AD

Note: The post hoc tests indicate statistically significant (*p* <.05) pairwise differences between patient groups, indicated by non-overlapping confidence intervals (CIs). For instance, DT > AD means the proportion among DT is statistically significantly larger than the proportion among AD, whereas DT > AD/AIP means that there is no statistically significant difference between AD and AIP but the proportions in these groups are smaller than that in DT

n.s.= not statistically significant

Furthermore, we analyzed whether all-cause mortality varied between DT and AD, as well as AIP and AD, while controlling for age and gender in Cox regression models with 95% CIs (see Table 3). Initially, we regressed mortality on the patient group, using age as the time variable. Subsequently, gender was included as a covariate in the analysis, which was justified given the absence of any interaction between gender and the diagnostic groups (AD/DT/AIP).

**Table 3:** Cox regression models for all-cause death by patient group. Hazard ratios (with 95% confidence intervals; CI) (*n* = 35 591)

	Alcohol dependence (AD)	Delirium tremens (DT)	Alcohol-induced psychosis (AIP)
Models	Hazard ratio	Hazard ratio (95% CI)	Hazard ratio (95% CI)
1) Adjusted for age	1.00 (ref)	1.57 (1.42–1.73)	0.99 (0.80–1.24)
2) Adjusted for gender and age	1.00 (ref)	1.53 (1.39–1.69)	1.00 (0.80–1.25)

The analyses were performed using SAS version 9.4. All patient data were fully deidentified for researchers, as required for information obtained from health registries where participant consent is not sought. Legal authorization and an exemption from confidentiality obligations for the use of personal data in research were provided by the Regional Committee for Medical and Health Research Ethics (2014/72/REK North).

## Results

The cohort consisted of three groups of patients; 33 107 patients received an AD diagnosis only, 700 patients received an AIP diagnosis, and 1 784 patients received a DT diagnosis (Table 1). Eighty-eight patients received diagnoses of both AIP and DT, of which 32 were included in the AIP group and 56 were included in the DT group. The average observation time from the index episode to censoring or December 31st, 2015, was 44.5 months for AD patients, 41.2 months for AIP patients, and 36.3 months for DT patients.

The AIP patients were youngest (mean 46.3 years; SD 14.3), and the DT patients oldest (56.0; 11.3) (*p* <.001). The DT patients were predominantly male (80.3% vs. 71.4 and 71.9%) (*p* <.001) and exhibited higher mortality both in the first month after the index episode (*p* <.001) and throughout the observation period (*p* <.001). The DT patients had the highest annual mortality rate, 8.0%, compared to 3.7% and 3.4% in AD and AIP patients, respectively. The Cox regression model showed that, compared to AD patients, only DT patients had an increased mortality when adjusting for age, which was not significantly altered by adjusting for gender (Table 3). This finding was also reflected in the Kaplan-Meier plots (Fig. 1).

Table 2 shows that DT patients exhibited significantly higher all-cause and natural cause SMRs (9.7 and 8.6 respectively) compared to both AD and AIP patients. DT patients differed from AD patients in all categories of natural and unnatural causes of death, except for respiratory disease and cancer. AIP patients, however, did not show any significant differences in causes of death compared to AD patients when examining SMRs. It is noteworthy that a somewhat larger proportion of AIP patients (6.2%) lacked a recorded cause of death, compared to AD (2.4%) and DT (1.8%) patients (data not shown in table).

Table 4 shows for each of the three patient groups, that the prevalence of all comorbidities was higher after the index episode than before. 42% of AIP had an AD diagnosis in the two-year period before the index episode, and 52.6% had an AD diagnosis in the two-year period after the index episode. Corresponding figures for DT were 50.4% and 64.4%. Alcohol-related comorbidities such as liver disease and polyneuropathy were more prevalent among DT patients before index episode, compared to AIP patients and AD patients. After the index episode this was true for more co-morbidities. For some of the comorbidities, there was a statistically significant difference between AD and AIP patients. This included acute alcohol intoxication, pancreatitis, and head injury prior to the index episode, and dementia after the index episode, where DT and AIP showed similar and elevated levels compared to patients with AD only. However, for those diseases mostly associated with chronic alcohol use, such as liver disease, pancreatitis, polyneuropathy, and head injuries, DT had the highest prevalence after the index episode. AIP and AD had comparable prevalence figures except for head injuries, where AIP showed prevalence figures between those of DT and AD patients. For schizophrenia spectrum disorders, and specifically for schizophrenia, AIP patients were in a unique position with much higher proportions both before and after the index episode compared to both AD and DT patients (Table 4).

**Table 4:** Presence of alcohol-related comorbidities prior to and after index episode by patient group (%)

	Comorbidities prior to episode					Comorbidities after episode				
	*N* (%)			*p*-value	Post hoc test	*N* (%)			*p*-value	Post hoc test
	Alcohol dependence (AD)	Delirium tremens (DT)	Alcohol-induced psychosis (AIP)			Alcohol dependence (AD)	Delirium tremens (DT)	Alcohol-induced psychosis (AIP)		
Alcohol intoxication	2 296 (6.9)	282 (15.8)	109 (15.6)	< 0.001	DT/AIP > AD <	2 845 (8.6)	372 (20.9)	121 (17.3)	< 0.001	DT > AIP > AD
Liver disease	939 (2.8)	148 (8.3)	19 (2.7)	< 0.001	DT > AD/AIP <	1 505 (4.5)	270 (15.1)	37 (5.3)	< 0.001	DT > AIP/AD
Pancreatitis	184 (0.6)	27 (1.5)	8 (1.1)	< 0.001	DT/AIP > AD	290 (0.9)	32 (1.8)	9 (1.3)	0.00	DT > AD
Polyneuropathy	427 (1.3)	55 (3.1)	8 (1.1)	< 0.001	DT > AD/AIP	670 (2.0)	101 (5.7)	13 (1.9)	< 0.001	DT > AIP/AD
Dementia	137 (0.4)	11 (0.6)	3 (0.4)	0.44	n.s	341 (1.0)	50 (2.8)	14 (2.0)	< 0.001	DT/AIP > AD
Schizophrenia spectrum (F2)	879 (2.7)	40 (2.2)	106 (15.1)	< 0.001	AIP > AD/DT	1 066 (3.2)	48 (2.7)	127 (18.1)	< 0.001	AIP > DT/AD
Schizophrenia (F20)	439 (1.3)	11 (0.6)	33 (4.7)	< 0.001	AIP > AD > DT	558 (1.7)	18 (1.0)	50 (7.1)	< 0.001	AIP > AD/DT
Head injury	2 637 (8.0)	246 (13.8)	76 (10.9)	< 0.001	DT/AIP > AD	2 538 (7.7)	265 (14.9)	78 (11.1)	< 0.001	DT > AIP > AD

Note: the *p*-values are given for the chi-square tests of the overall distribution. The post hoc tests indicate statistically significant (*p* <.05) pairwise differences between patient groups. For instance, AIP < DT means the proportion among DT is statistically significantly larger than the proportion among AIP, whereas AD < DT/AIP means that there is no statistically significant difference between DT and AIP but the proportions in these groups are larger than that in AD. n.s.= not statistically significant

## Discussion

In this register-based study, AIP patients were younger than the average AD patients, whereas DT patients were older. The gender representation was similar among AIP and AD patients, while DT patients had a higher representation of males. After adjusting for age and for gender, there was no increased mortality among individuals with AIP compared to AD patients, whereas DT patients exhibited increased mortality. AIP patients did not show increased cause-specific mortality compared to AD patients across a wide range of causes of death, whereas several cause-specific mortalities were higher among DT patients compared to AD patients. Comorbidity before and after the index episodes was quite similar for AIP patients and AD patients regarding diseases known to be associated with AD, like liver disease, pancreatitis, and polyneuropathy. The exception was schizophrenia spectrum disorders, where patients with AIP had a much higher prevalence both before and especially after the index episode. DT patients had a higher prevalence of most comorbid disorders before and after the index episode compared to patients with AD or AIP.

The younger age among the AIP patients may reflect a more typical age of onset of psychotic disorders, which is between 15 and 30 years [31], while the typical age for seeking treatment for AD is much higher [32]. The higher age for those with DT aligns with previous findings and may indicate that these represent cases of more long-lasting, and perhaps more severe, AD [20, 33]. Given these differences in age and gender, it was important to present data either adjusted for age or as standardized measures. We were not able to adjust for socioeconomic status due to limitations in the data.

The AIP patients demonstrated no increased cause-specific mortality compared to AD patients across a wide range of causes of death, while several cause-specific mortalities were higher among DT patients. This latter finding is consistent with earlier research on DT [14, 20], and supports the view that the DT patients can be considered the more severe cases of AD. We know that alcohol use is negatively related to more than 200 different conditions and diseases [34, 35]. Of special note, we even observed a greater SMR for cardiovascular disease, in line with earlier research pointing to heavy alcohol use being a risk factor for this [36].

Concerning comorbidity before and after the index episodes, AIP patients were quite similar to AD patients regarding somatic diseases known to be associated with AD, such as liver disease, pancreatitis, and polyneuropathy. DT patients had a higher prevalence of most comorbid disorders compared to both AIP and AD, both before and after the index episode, again possibly reflecting that the DT patients were older and represent the more severe cases of AD. The exception for AIP patients were the schizophrenia spectrum disorders, where AIP had a much higher prevalence both before and especially after the index episode. This is of interest due to the ongoing discussion about how AIP should be viewed in relation to schizophrenia [37]. Here, views differ from considering AIP as a condition that shares many similarities with schizophrenia spectrum disorders, both etiologically and clinically [24], to viewing it as a malady distinct from schizophrenia, despite the similarities in symptoms [2, 3, 23]. We have recently published a study indicating that one in ten AIP patients over a six-year period are diagnosed with a schizophrenia spectrum disorder [25]. This and the current findings support the notion that AIP should be viewed as not only a phenomenon distinctly separate from DT, but also a clinical phenomenon closely related to schizophrenia. Even if AIP and schizophrenia are separate diagnoses and by some are viewed as separate phenomena, the current study indicates a proximity to schizophrenia spectrum disorders. This could be viewed as a challenge for the nosological validity of the AIP diagnosis. One could further argue that instead of calling it AIP, we should refer to it as first episode psychosis among alcohol users, like we have earlier suggested for cannabis and amphetamine induced psychosis [37]?

## Limitations

This study used data from two comprehensive Norwegian health registries: The NPR and the NCDR. However, our findings may be subject to bias. Many individuals with AUDs are not treated in specialized care, leading to an overrepresentation of severe cases in our data and potentially, inflated mortality and comorbidity rates [38, 39]. Conversely, specialized care might have been more accessible for patients with AIP and DT due to the more acute nature of these conditions compared with AD, possibly reducing the effect sizes found. Additionally, diagnoses are taken at face value and are solely based on the attending physician’s assessment without external review, potentially introducing inaccuracies. To the best of our knowledge no validation studies on the diagnosis found in NPR have been performed. Finally, because we only tracked patients for a limited time, our understanding of long-term morbidity and mortality is incomplete.

On the other hand, this study is based on a large sample and investigates morbidity and mortality for individuals with AIP, DT and AD in a way that has not previously been done.

## Conclusion

In the current observational study utilizing Norwegian patient registry data we had no preregistration and can also not draw any conclusion on causality. Our findings indicate that AIP patients have a mortality and morbidity comparable to other AD patients. This is different from patients with DT who have a higher morbidity and mortality. However, it is of note that patients with an AIP had higher prevalence of schizophrenia spectrum disorder and schizophrenia.

## Data Availability

Availability of data and materials: The data that support the findings of this study are available from the Norwegian Patient Registry (NPR) but restrictions apply to the availability of these data, which were used under license for the current study, and so are not publicly available. Data are however available from the authors upon reasonable request and with permission of NPR.

## Declarations

### Disclaimer

Data from the Norwegian Patient Registry and the Norwegian Cause of Death Registry have been used in this publication. The interpretation and reporting of these data are the sole responsibility of the authors, and no endorsement by any of these registries is intended nor should be inferred.

## Abbreviations

AD: Alcohol dependency
AIP: Alcohol-induced psychosis
AUD: Alcohol use disorder
CI: Confidence interval
DT: Delirium tremens
NCDR: Norwegian Cause of Death Registry
NPR: Norwegian Patient Registry
SMR: Standardized mortality ratio
